# Supplementary material for: Sensitive neoantigen discovery by real-time mutanome-guided immunopeptidomics
Source: Nat Commun. 2025 Aug 7;16:7269. doi: 10.1038/s41467-025-62647-4 (PMC12332187; doi:10.1038/s41467-025-62647-4)
Supplement: Supplementary file 1 — Supplementary Information [file 41467_2025_62647_MOESM1_ESM.pdf]

# Sensitive neoantigen discovery by real-time mutanome-guided immunopeptidomics

Ilja E Shapiro[1,2,3], Florian Huber[1,2,3], Justine Michaux[1,2,3], Michal Bassani-Sternberg*[1,2,3]

[1]Department of Oncology, University of Lausanne (UNIL) and Lausanne University Hospital (CHUV), Lausanne, (Vaud) Switzerland

[2]Ludwig Institute for Cancer Research, Lausanne Branch, Lausanne, (Vaud) Switzerland

[3]Agora Cancer Research Centre, 1011 Lausanne, Switzerland.

* Corresponding author: michal.bassani@chuv.ch

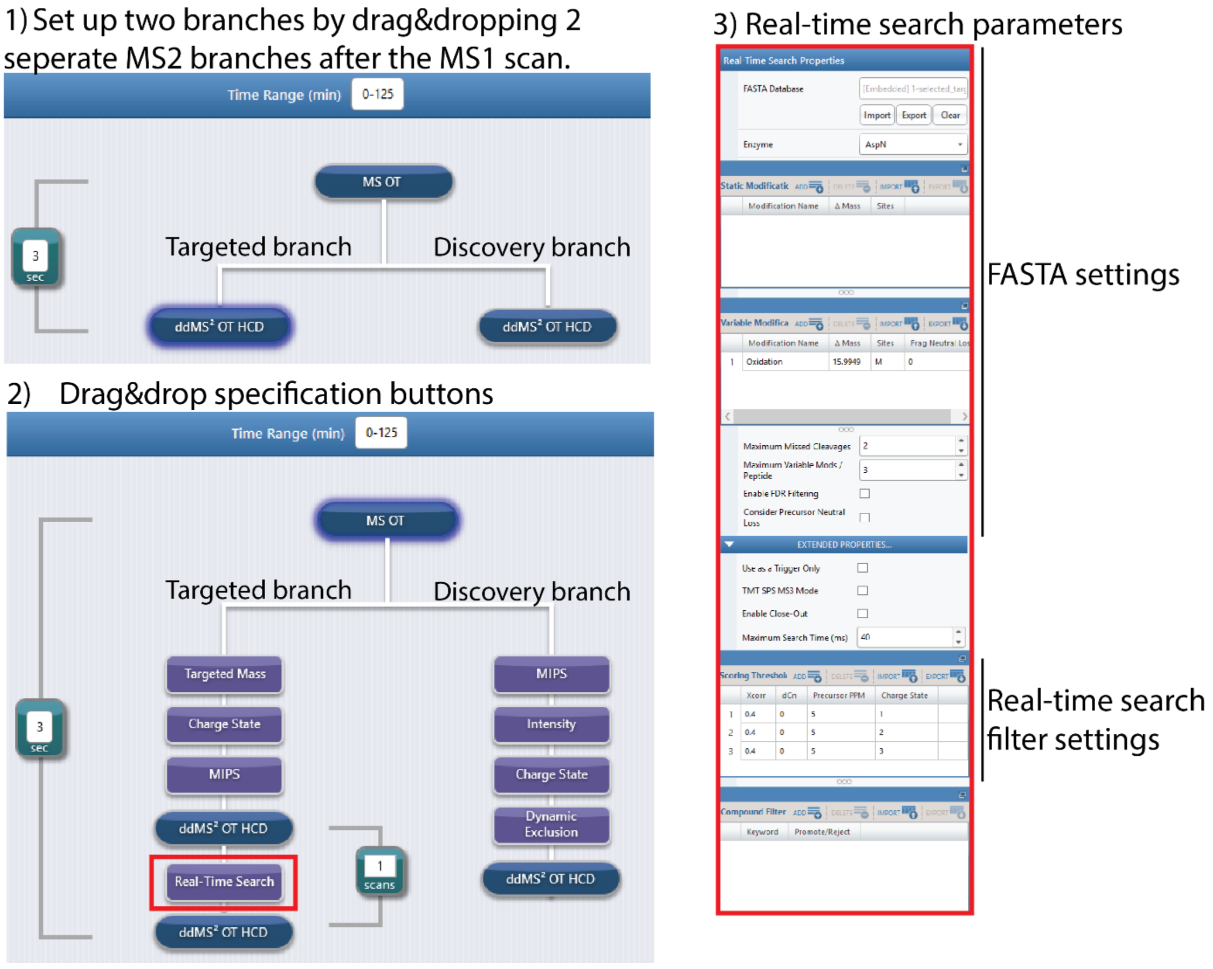


**Suppl. Fig. 1: NeoDiscMS method set-up.** NeoDiscMS implementation applies real-time search functionality and was designed for ease-of-use, requiring few steps for method set-up. Step 3 shows the parameters sidebar when clicking the “Real-time search” button in step 2.

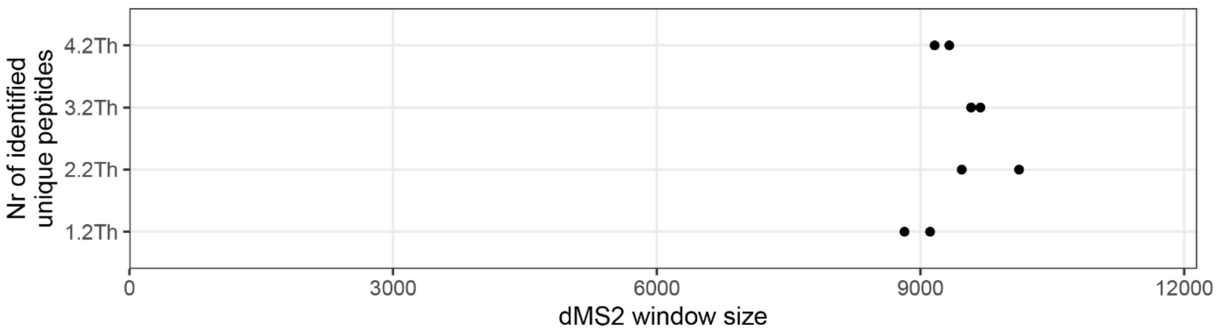


**Suppl. Fig. 2: Comparison of different MS2 window sizes when processing with MSFragger-DDA+**. Number of uniquely identified peptides for DDA acquisitions of 10 million JY cells equivalent / injection (n = 2).

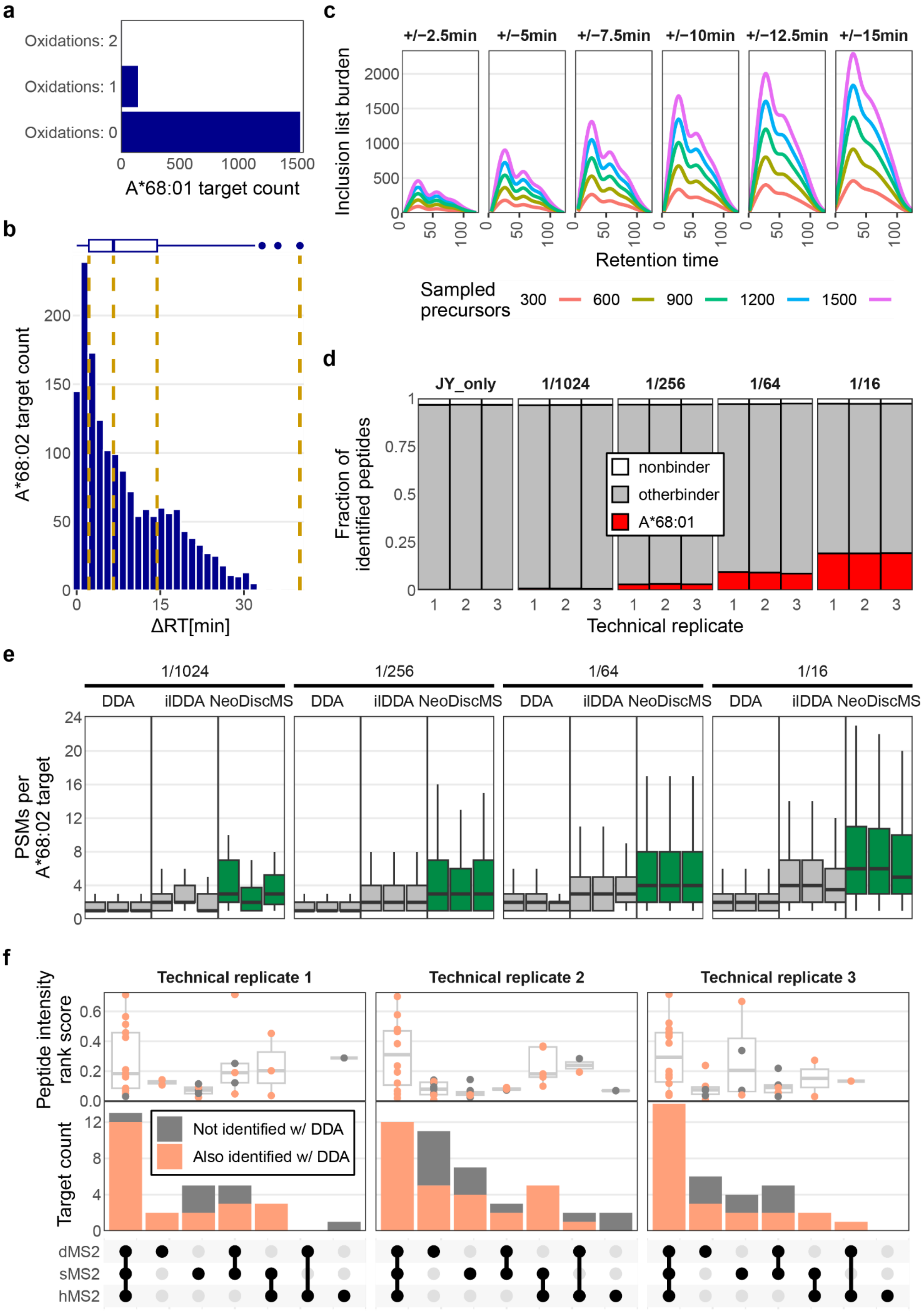

a
Oxidations: 2
Oxidations: 1
Oxidations: 0
0 500 1000 1500
A*68:01 target count
b
A*68:02 target count
ΔRT[min]
c
+/−2.5min +/−5min +/−7.5min +/−10min +/−12.5min +/−15min
Inclusion list burden
Retention time
Sampled precursors 300 600 900 1200 1500
d
JY_only 1/1024 1/256 1/64 1/16
Fraction of identified peptides
nonbinder
otherbinder
A*68:01
Technical replicate
e
1/1024 1/256 1/64 1/16
DDA ilDDA NeoDiscMS
PSMs per A*68:02 target
f
Technical replicate 1
Technical replicate 2
Technical replicate 3
Peptide intensity rank score
Target count
Not identified w/ DDA
Also identified w/ DDA
dMS2
sMS2
hMS2

**Suppl. Fig. 3: HLA-A*68:01 target selection and identification with DDA, ilDDA, and NeoDiscMS in a dilution series of the RA957 immunopeptidome diluted in the JY immunopeptidome**. **a)** Number of target peptides after considering methionine oxidation as a variable post-translational modification. **b)** Histogram of the absolute time difference between measured and predicted retention time (x-axis). **c)** After RT prediction for each target peptide, we estimated the number of precursors to be included in the inclusion list during chromatographic separation. We randomly sample 300, 600, 900, 1200, or 1500 precursors 100 times to estimate the average inclusion list burden at any moment during the RT. **d)** Fraction of HLA-A*68:01 binders in the different JY samples spiked with dilution series of RA957 immunopeptidome (n = 3 per condition), identified from DDA data. **e)** Number of PSMs per target peptide found with DDA, ilDDA, or NeoDiscMS. **f)** Target peptide identifications are made with all scan types. dMS2, sMS2, and hMS2 scans together provide a diversity that makes measurements more sensitive to targets while also reducing the stochasticity DDA is subjected to. Whisker length indicates 1.5*IQR (interquartile range).

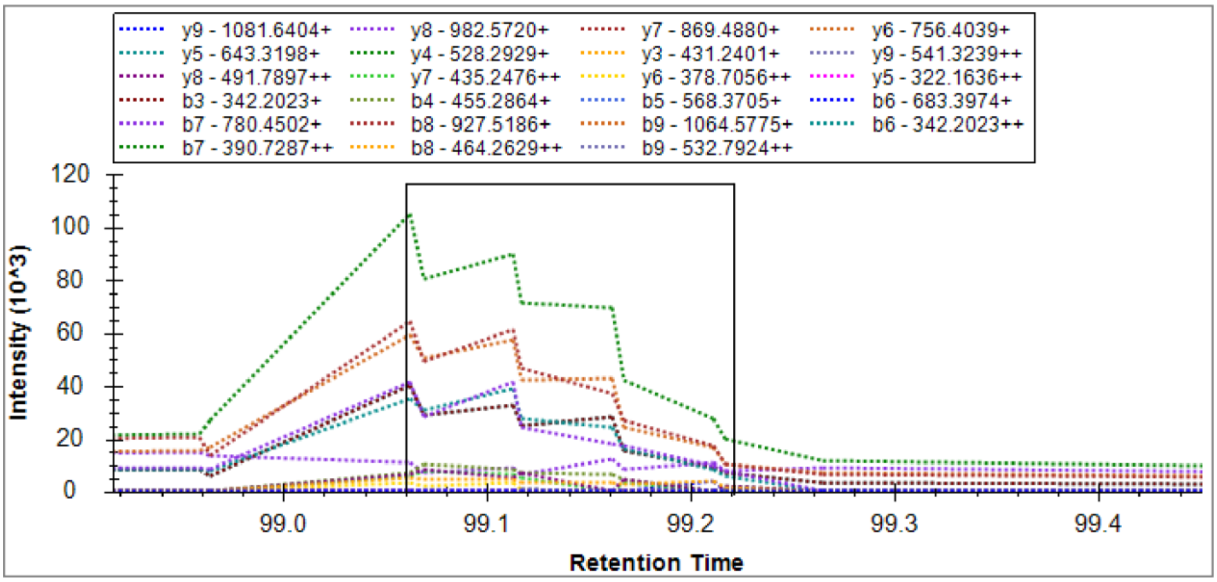


**Suppl. Fig. 4: Fragment ion currents of EVILIDPFHK++ with different MS2 scan types**. Elution profile of target EVILIDPFHK++ as acquired by NeoDiscMS (1=1024 dilution, technical replicate 1), the black box indicates the interval where 7 PSMs were identified within 11 seconds.

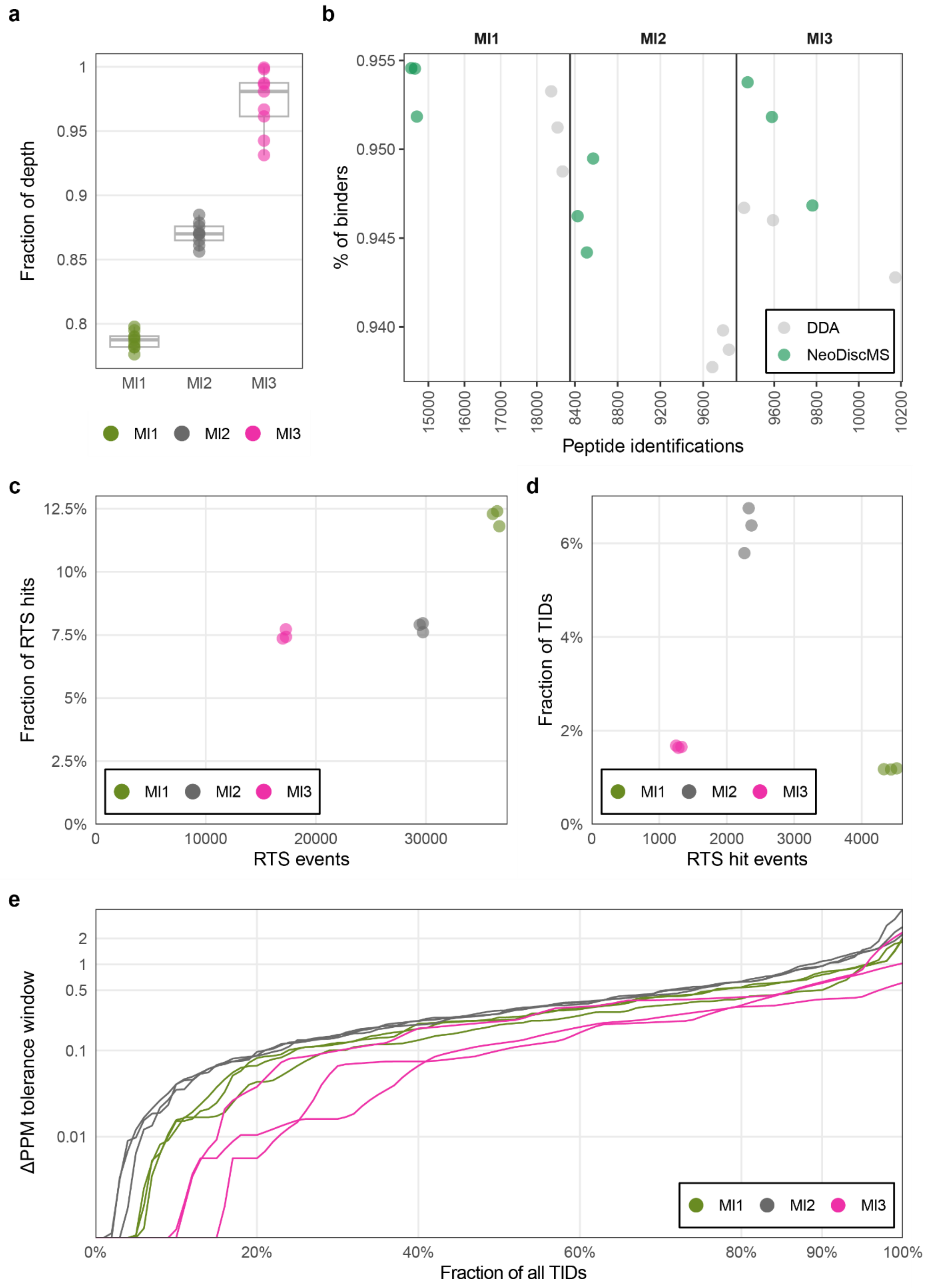
a
b
Fraction of depth
1
0.95
0.9
0.85
0.8
MI1
MI2
MI3
MI1
MI2
MI3
% of binders
0.955
0.950
0.945
0.940
15000
16000
17000
18000
8400
8800
9200
9600
9600
9800
10000
10200
Peptide identifications
DDA
NeoDiscMS
c
d
Fraction of RTS hits
12.5%
10%
7.5%
5%
2.5%
0%
0
10000
20000
30000
RTS events
Fraction of TIDs
6%
4%
2%
0%
1000
2000
3000
4000
RTS hit events
e
ΔPPM tolerance window
2
1
0.5
0.1
0.01
0%
20%
40%
60%
80%
90%
100%
Fraction of all TIDs

**Suppl. Fig. 5: Sample quality, loss in global depth when comparing NeoDiscMS to DDA, and RTS hit characterization for Ml1, Ml2, and Ml3. a)** Loss in global depth with respect to identified unique peptides with NeoDiscMS compared to DDA (n = 3 per condition). Three raw files each, per cell line model, yields 9 comparisons each. Whisker length indicates 1.5*IQR (interquartile range). **b)** Comparison of samples and replicates regarding the number of identified unique peptides and the percentage of binders (rank < 2% with MixMHCpred). **c)** Percentage of scans triggered by the scheduled inclusion list (sMS2) that lead to passing real-time search filters. **d)** Fraction of instances where an hMS2 is triggered (RTS hit event), leads to the identification of the isolated precursor as the target (TID). **e)** Each line is a NeoDiscMS measurement and colours indicate the cell line model. Lines indicated what fraction of all TIDs fit into the smallest possible mass deviation (determined by RTS) window.

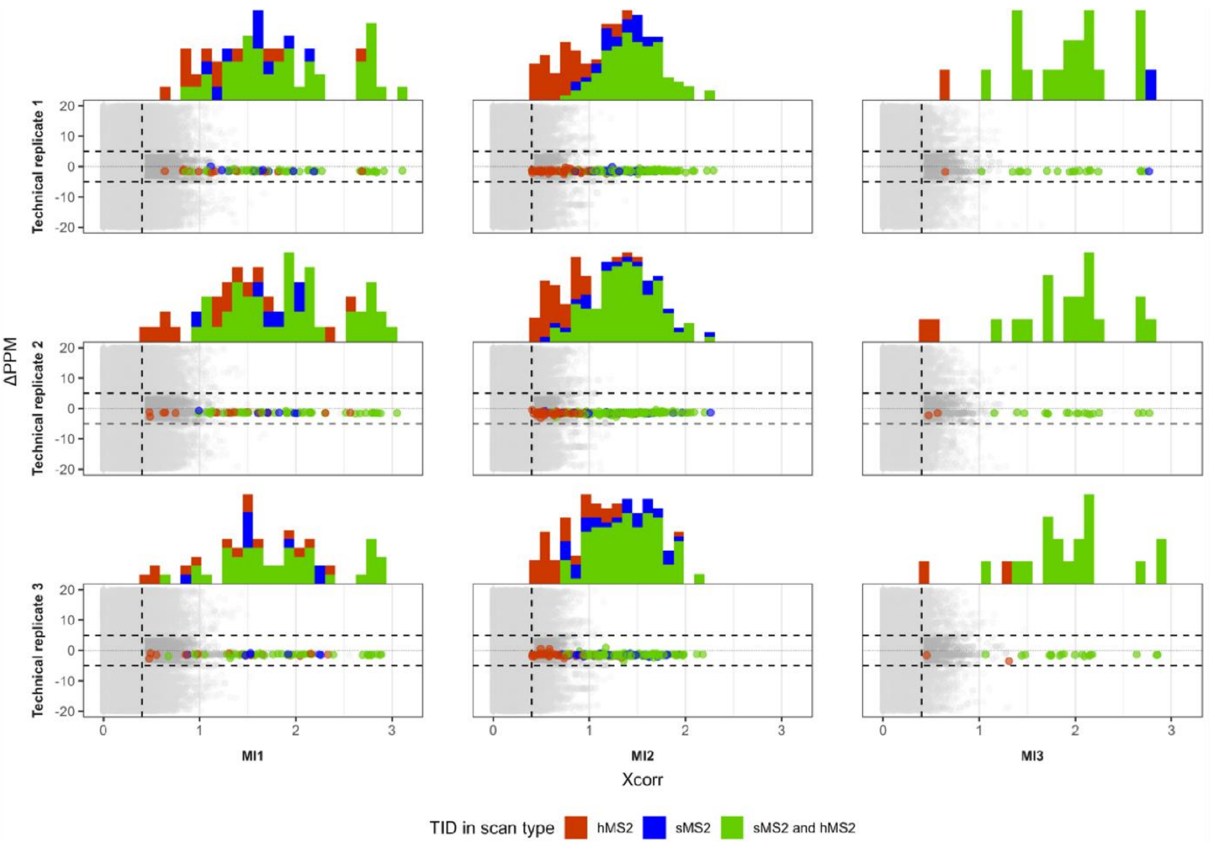


**Suppl. Fig. 6: Real-time search cross-correlation (Xcorr) and precursor mass deviation (ΔPPM) values provide insights into the acquisition dynamics and efficiency of NeoDiscMS.** For example, they indicate the cross-correlation values that lead to target identifications and highlight where these occur exclusively for hMS2, but not for the corresponding sMS2.

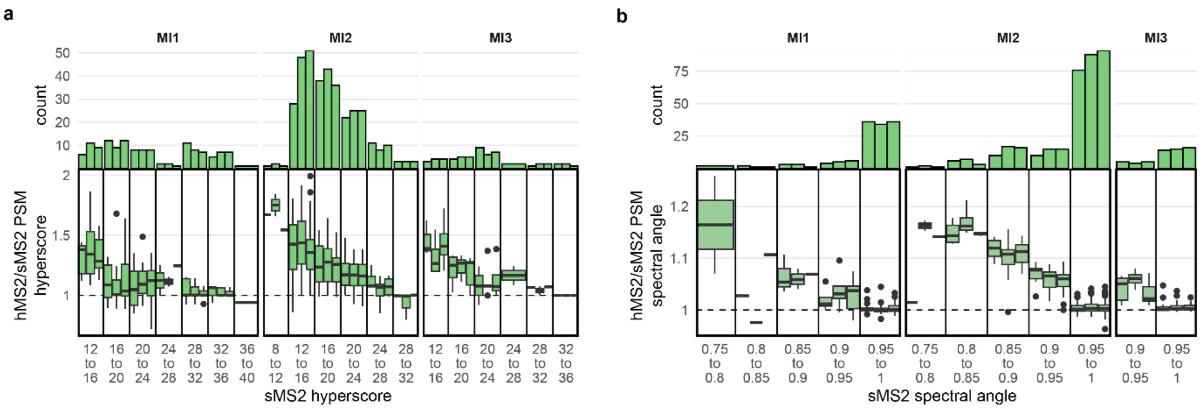


**Suppl. Fig. 7: Change is PSM quality between target identifications made with both sMS2 and hMS2 of the same RTS event. a)** The lower panel shows changes in hyperscore, with bins of sMS2 hyperscore ranges (x-axis) and the fold-change (y-axis) when comparing hMS2 hyperscores to sMS2 hyperscores. The upper panel shows the absolute number of RTS event in each bin. Replicates (n = 3 per conditions) are each visualized separately. **b)** Same structure as in a), but spectral angles and not hyperscores. Whisker length indicates 1.5*IQR (interquartile range).

| | dMS2 | sMS2 | hMS2 |
|---|---|---|---|
| Used in method(s) | DDA, iIDDA, NeoDiscMS | NeoDiscMS | iIDDA, NeoDiscMS |
| Purpose of scan | Global coverage | RTS assessment of precursor scan | High sensitivity scan |
| Scan trigger | TopN | Inclusion list | Inclusion list (iIDDA), RTS filters (NeoDiscMS) |
| Isolation window [Da] | 3.2 | 1.2 | 1.2 |
| Max. Inj. Time [ms] | 54 | 54 | 250 |
| Max. AGC [%] | 100 | 100 | 300 |
| Min. peak intensity for fragmentation [IC] | 1.00E+05 | - | - |
| Normalized collision energy [%] | 30 | 30 | [27,30,32] |
| Dynamic exclusion | Yes | No | No |
| Avoid fragmenting multiple charge states of same precursor | Yes | No | No |

**Suppl. Table 1:** Key acquisition parameters of dMS2, sMS2, and hMS2 scan types.

| Name | Cancer | Sample type | HLA I typing |
|---|---|---|---|
| Ml1 | melanoma | cell line | A*02:05, A*32:01, B*07:02, B*44:03, C*04:01, C*07:02 |
| Ml2 | melanoma | cell line | A*24:02, A*26:01, B*35:03, B*38:01, C*12:03 |
| Ml3 | melanoma | cell line | A*01:01, A*03:01, B*08:01, B*57:01, C*06:02, C*07:01 |
| Ti1 | melanoma | tissue | A*02:01, A*11:01, B*14:01, B*35:01, C*04:01, C*08:02 |
| Ti2 | melanoma | tissue | A*02:01, A*11:01, B*14:01, B*35:01, C*04:01, C*08:02 |
| Ti3 | melanoma | tissue | A*02:01, A*11:01, B*14:01, B*35:01, C*04:01, C*08:02 |

**Suppl. Table 2:** Class I HLA-typing of Ml1, Ml2, Ml3, Ti1, Ti2, and Ti3.

| Scan type | Mass resolution | Mass range [Th] | Isolation window[Th] | AGC target[%] | Max injection time[ms] | NCE |
|---|---|---|---|---|---|---|
| MS1 | 120000 | 300-1650 | - | 100 | 50 | - |
| dMS2-1p2 | 30000 | - | 1.2 | 100 | 54 | 30 |
| dMS2-3p2 | 30000 | - | 3.2 | 100 | 54 | 30 |
| sMS2 | 30000 | - | 1.2 | 100 | 54 | 30 |
| hMS2 | 30000 | - | 1.2 | 300 | 250 | 27,30,32 |

**Suppl. Table 3:** Table with scan type parameters.
