# Supplementary material for: Sensitive neoantigen discovery by real-time mutanome-guided immunopeptidomics
Source: Nat Commun. 2025 Aug 7;16:7269. doi: 10.1038/s41467-025-62647-4 (PMC12332187; doi:10.1038/s41467-025-62647-4)
Supplement: Supplementary file 3 — Reporting Summary [file 41467_2025_62647_MOESM3_ESM.pdf]

nature portfolio

Corresponding author(s): Michal Bassani-Sternberg

Last updated by author(s): Jan 29, 2025

Reporting Summary

Nature Portfolio wishes to improve the reproducibility of the work that we publish. This form provides structure for consistency and transparency in reporting. For further information on Nature Portfolio policies, see our Editorial Policies and the Editorial Policy Checklist.

Statistics

For all statistical analyses, confirm that the following items are present in the figure legend, table legend, main text, or Methods section.

n/a	Confirmed	
☐	☒	The exact sample size (*n*) for each experimental group/condition, given as a discrete number and unit of measurement
☐	☒	A statement on whether measurements were taken from distinct samples or whether the same sample was measured repeatedly
☒	☐	The statistical test(s) used AND whether they are one- or two-sided<br>*Only common tests should be described solely by name; describe more complex techniques in the Methods section.*
☒	☐	A description of all covariates tested
☐	☒	A description of any assumptions or corrections, such as tests of normality and adjustment for multiple comparisons
☐	☒	A full description of the statistical parameters including central tendency (e.g. means) or other basic estimates (e.g. regression coefficient) AND variation (e.g. standard deviation) or associated estimates of uncertainty (e.g. confidence intervals)
☒	☐	For null hypothesis testing, the test statistic (e.g. *F*, *t*, *r*) with confidence intervals, effect sizes, degrees of freedom and *P* value noted<br>*Give P values as exact values whenever suitable.*
☒	☐	For Bayesian analysis, information on the choice of priors and Markov chain Monte Carlo settings
☒	☐	For hierarchical and complex designs, identification of the appropriate level for tests and full reporting of outcomes
☒	☐	Estimates of effect sizes (e.g. Cohen's *d*, Pearson's *r*), indicating how they were calculated

Our web collection on statistics for biologists contains articles on many of the points above.

Software and code

Policy information about availability of computer code

Data collection	No software was used for data collection.
Data analysis	Softwares used in this study are publicly available and stated in the Methods section where applicable.<br>-NeoDisc v1.7.0<br>-MS analyses: PDV 1.7.4, FragPipe v22.0<br>-MixMHCpred v2.3<br>-General: R v4.4

For manuscripts utilizing custom algorithms or software that are central to the research but not yet described in published literature, software must be made available to editors and reviewers. We strongly encourage code deposition in a community repository (e.g. GitHub). See the Nature Portfolio guidelines for submitting code & software for further information.

Data

Policy information about availability of data

All manuscripts must include a data availability statement. This statement should provide the following information, where applicable:

- Accession codes, unique identifiers, or web links for publicly available datasets
- A description of any restrictions on data availability
- For clinical datasets or third party data, please ensure that the statement adheres to our policy

The mass spectrometry immunopeptidomics raw files, reference fasta files and NewAnce, Fragpipe and Spectronaut parameters and outputs have been deposited to the ProteomeXchange Consortium via the PRIDE partner repository with the dataset identifier PXD059824.

Human research participants

Policy information about studies involving human research participants and Sex and Gender in Research.

Reporting on sex and gender	Male/female information was collected based on informed consent. This was a proof-of-concept exploratory study with a small set of samples. Sex and gender were not considered in the study design. No analysis or correlations based on sex and gender were performed.
Population characteristics	Not applicable.
Recruitment	Tissues from one patient were collected and biobanked. This patient was selected based on sample availabitliy. Material was enough to conduct immunopeptidomics. This selection should not have any impact on the results obtained.
Ethics oversight	An informed consent was given by the participants, according to the requirements of the institutional review board (Ethics Commission, CHUV).

Note that full information on the approval of the study protocol must also be provided in the manuscript.

Field-specific reporting

Please select the one below that is the best fit for your research. If you are not sure, read the appropriate sections before making your selection.

[x] Life sciences [ ] Behavioural & social sciences [ ] Ecological, evolutionary & environmental sciences

For a reference copy of the document with all sections, see nature.com/documents/nr-reporting-summary-flat.pdf

Life sciences study design

All studies must disclose on these points even when the disclosure is negative.

Sample size	For comparison of LC-MS data acquisition method a sample size of three per condition is sufficient to distuingish characteristics between methods.
Data exclusions	No data was excluded
Replication	We compare NeoDiscMS to DDA in multiple settings with different experimental models.
Randomization	The only room for randomization for this project was the order in which data was acquired for a given experiment, which we did randomize.
Blinding	Blinding was not relevant to our study, as we did not process samples within an experiment differently. Our conditions were based on different data acquisition modes for the same samples.

Reporting for specific materials, systems and methods

We require information from authors about some types of materials, experimental systems and methods used in many studies. Here, indicate whether each material, system or method listed is relevant to your study. If you are not sure if a list item applies to your research, read the appropriate section before selecting a response.

Materials & experimental systems

n/a	Involved in the study	
☐	☒	Antibodies
☐	☒	Eukaryotic cell lines
☒	☐	Palaeontology and archaeology
☒	☐	Animals and other organisms
☒	☐	Clinical data
☒	☐	Dual use research of concern

Methods

n/a	Involved in the study	
☒	☐	ChIP-seq
☒	☐	Flow cytometry
☒	☐	MRI-based neuroimaging

Antibodies

Antibodies used

W6/32 antibodies, produced by HB-95 hybridoma cells (ATCC® HB-95™)
Company name : ATCC
Catalog number : HB-95
Lot number: 7001294
Clone name: W6/32
Antigenic determinant: HLA-A, B, C
Isotype: IgG2a
Host: mouse
Cell type: Hybridoma: B lymphocyte
Clonality: monoclonal

Validation

Validation by vendor following ATCC guidelines. Certificate of Analysis can be found here: https://www.lgcstandards-atcc.org/Products/All/HB-95.aspx?geo_country=ch#documentation

Additionally, anti-HLA-I antibody was validated directly in our laboratory, through the use of this antibody for immuno-affinity purification of HLA-I peptides from cell lines and tissue samples. These peptides were measured by mass spectrometry, and their characteristics fit that of HLA-I peptides.

Eukaryotic cell lines

Policy information about cell lines and Sex and Gender in Research

Cell line source(s)	Melanoma cell lines were provided by the Center of Experimental Therapy Biobank (CHUV), Lausanne. Approving committee was the Commission cantonale d'éthique de la recherche sur l'être humain (CER-VD).
Authentication	Samples were authenticated by comparing the names labelled on the vials received with the providers' information. Molecular HLA typing was performed.
Mycoplasma contamination	Cells were not tested for microplasma contamination
Commonly misidentified lines (See ICLAC register)	No commonly misidentified cell lines were used in the study
