# Supplementary material for: Sensitive neoantigen discovery by real-time mutanome-guided immunopeptidomics
Source: Nat Commun. 2025 Aug 7;16:7269. doi: 10.1038/s41467-025-62647-4 (PMC12332187; doi:10.1038/s41467-025-62647-4)
Supplement: Supplementary file 4 — Description of Additional Supplementary Files [file 41467_2025_62647_MOESM4_ESM.pdf]

Description of Additional Supplementary Files

File name: Supplementary Data 1

Description: List of LC-MS experiments, sample input material, and raw fle names.

File name: Supplementary Data 2

Description: TAA identifrs and sequences used for in silico three-frame stop-to-stop translaton.

File name: Supplementary Data 3

Description: Example for a DeepLC calibraton peptdes input.

File name: Supplementary Data 4

Description: Example for a DeepLC target pepties input.

File name: Supplementary Data 5

Description: Example for a scheduled inclusion list.

File name: Supplementary Data 6

Description: Example for a target peptie FASTA database.
